# Real-World Evaluation of Quality of Life, Effectiveness, and Safety of Aflibercept Plus FOLFIRI in Patients with Metastatic Colorectal Cancer: The Prospective QoLiTrap Study

**DOI:** 10.3390/cancers14143522

**Published:** 2022-07-20

**Authors:** Ralf-Dieter Hofheinz, Sandro Anchisi, Birgit Grünberger, Hans G. Derigs, Mark-Oliver Zahn, Christine Geffriaud-Ricouard, Max Gueldner, Christine Windemuth-Kieselbach, Stefanie Pederiva, Pierre Bohanes, Felicitas Scholten, Gudrun Piringer, Josef Thaler, Roger von Moos

**Affiliations:** 1 Department of Oncology, University Hospital Mannheim, Theodor-Kutzer-Ufer 1, 68167 Mannheim, Germany; 2 Department of Oncology, Valais Romand Hospital Center, Valais Hospital, Av. Grand-Champsec 86, 1951 Sion, Switzerland; sandro.anchisi@hopitalvs.ch; 3 Department of Internal Medicine, Hematology and Oncology, Hospital Wiener Neustadt, Corvinusring 3-5, 2700 Wiener Neustadt, Austria; birgit.gruenberger@wienerneustadt.lknoe.at; 4 Department of Internal Medicine 3, Hematology, Oncology, Palliative Medicine and Pneumology, Frankfurt Höchst Clinic, Gotenstrasse 6-8, 65929 Frankfurt am Main, Germany; derigs@klinikumfrankfurt.de (H.G.D.); felicitas.scholten@klinikumfrankfurt.de (F.S.); 5 Department of Internal Medicine, Hematology and Oncology, MVZ Oncology Cooperation Harz, Kosliner Str. 14, 38642 Goslar, Germany; m-o.zahn@onkologie-goslar.de; 6 Sanofi, Global Medical Oncology, 54 Rue La Boetie, 75008 Paris, France; christine.geffriaud-ricouard@sanofi.com; 7 Sanofi, Potsdamer Str. 8, 10785 Berlin, Germany; max.gueldner@sanofi.com; 8 Department of Biometry, Alcedis, Winchesterstr 3, 35394 Giessen, Germany; cwk@alcedis.de; 9 Center for Oncology and Hematology, Cantonal Hospital, Im Ergel, 5404 Baden, Switzerland; stefanie.pederiva@ksb.ch; 10 Department of Oncology and Internal Medicine, Center for Chemotherapy, 1004 Lausanne, Switzerland; pierre.bohanes@ccac.ch; 11 Department of Internal Medicine IV, Wels-Grieskirchen Hospital, Grieskirchner Str. 42, 4600 Wels, Austria; gudrun.piringer@hotmail.com (G.P.); josef.thaler@klinikum-wegr.at (J.T.); 12 Johannes Kepler University Linz, Altenberger Strasse 69, 4040 Linz, Austria; 13 Department of Oncology, Cantonal Hospital Graubuenden, Loestrasse 170, 7000 Chur, Switzerland; roger.vonmoos@ksgr.ch

**Keywords:** colorectal cancer, aflibercept, VEGF, EGFR inhibitors, quality of life, anti-angiogenics

## Abstract

**Simple Summary:**

Colorectal cancer (CRC) is the third most frequently diagnosed malignancy worldwide and the second leading cause of cancer-related mortality. In VELOUR phase III trial, aflibercept combined with FOLFIRI has been shown to prolong overall survival versus FOLFIRI plus placebo in metastatic CRC (mCRC) patients previously treated with an oxaliplatin-based regimen. However, VELOUR did not evaluate patient quality of life and, outcomes following treatment with epidermal growth factor receptor inhibitors (EGFR-I) were unknown. In this prospective study conducted in daily practice, mCRC patients treated with aflibercept plus FOLFIRI maintained their quality of life as assessed by the EORTC QLQ-C30 questionnaire. Aflibercept plus FOLFIRI was also associated with a high objective tumor response and retained its activity regardless of sex, *RAS* status, and prior targeted therapy, especially after EGFR-I. Adverse events were consistent with the known safety profile of aflibercept plus FOLFIRI.

**Abstract:**

Aflibercept plus FOLFIRI prolongs overall survival (OS) in patients with metastatic colorectal cancer after the failure of oxaliplatin-containing therapy. QoLiTrap prospectively evaluated the quality of life (QoL) and effectiveness of this regimen in daily clinical practice, according to *RAS* status, sex, and prior targeted therapy, especially epidermal growth factor receptor inhibitors (EGFR-I). The primary endpoint was the percentage of patients whose EORTC QLQ-C30 global health status (GHS) improved or reduced by <5% from baseline during the first 12 weeks of therapy. Secondary endpoints included objective response rate (ORR), progression-free survival (PFS), overall survival (OS), and safety. One thousand two hundred and seventy-seven patients were treated with aflibercept plus FOLFIRI and 872 were evaluable for QoL. GHS improved or decreased by <5% in 40.3% of cases. The ORR was 20.8%, the median PFS was 7.8 months (95% confidence interval (CI), 7.3–8.3), and the median OS was 14.4 months (95% CI, 13.1–18.1). After prior EGFR-I, the ORR was 23.7%, median PFS was 9.4 months (95% CI, 6.5–12.9), and median OS was 17.4 months (95% CI, 10.5–33.7). The safety profile was consistent with previously reported data. Aflibercept plus FOLFIRI given in daily practice maintained QoL in mCRC patients, was associated with a high objective tumor response, and retained its activity regardless of sex, *RAS* status, and prior EGFR-I therapy.

## 1. Introduction

Colorectal cancer (CRC) is the third most frequently diagnosed malignancy worldwide and the second leading cause of cancer-related mortality [1,2]. Generally, the risk of CRC increases with age [3,4]. However, in the last decade, incidence rates in individuals aged >55 years have decreased or remained stable, whereas rates have increased in young adults aged 20–39 years [5]. If detected early, patients with CRC have a 5-year overall survival (OS) rate of about 90%, but in cases of metastatic CRC (mCRC), the prognosis is poor with a 5-year OS of only 13% [3].

Current treatment options for patients with mCRC are limited. Resection of metastases is the only treatment that currently offers a chance of long-term OS [6,7]. The primary goal of treatment, therefore, is to increase a patient’s likelihood of successful metastatic resection [8,9]. In fit patients in whom cytoreduction is the goal, the first-line recommendation is treatment with a cytotoxic doublet plus epidermal growth factor receptor inhibitors (EGFR-I) for *RAS* wild-type tumors of the left colon, and a cytotoxic doublet or triplet (for suitable patients) plus bevacizumab for *RAS* mutant tumors or *RAS* wild-type tumors of the right colon or *BRAF* mutant tumors [8]. Patients harboring a primary tumor with microsatellite instability should receive treatment with checkpoint inhibitors [8]. For second-line therapy, guidelines recommend changing the chemotherapy backbone and continuing or switching the anti-angiogenic agent if the tumor is *RAS* mutant [8]. For patients with *RAS* wild-type tumors, guidelines recommend anti-angiogenic treatment [8].

Aflibercept is a recombinant fusion protein that blocks the activity of vascular endothelial growth factors (VEGF)-A, (VEGF)-B, and placental growth factor (PlGF) by acting as a high-affinity ligand trap, thereby preventing these ligands from binding to their endogenous receptors [10,11]. In a randomized, placebo-controlled phase III trial (VELOUR), aflibercept combined with FOLFIRI (fluorouracil [5-FU], leucovorin, irinotecan) significantly prolonged OS (13.5 vs. 12.0 months, hazard ratio [HR], 0.817; 95% CI, 0.713–0.937; *p* = 0.0032) and progression-free survival (PFS) (6.9 vs. 4.7 months, HR, 0.758; 95% CI, 0.661–0.869; *p* < 0.0001) versus FOLFIRI alone [12]. In addition, despite the enrollment of early progressors after first-line treatment in VELOUR, aflibercept plus FOLFIRI was associated with a response rate almost twice as high as that with FOLFIRI alone (19.8% vs. 11.1%, *p* = 0.0001) [12]. Based on these results, aflibercept plus FOLFIRI was approved in the United States in August 2012 and in the European Union in March 2013 for the treatment of patients with mCRC who are resistant to or have progressed following an oxaliplatin-containing regimen.

However, several clinically important questions remained unanswered by the VELOUR study. First, the impact of aflibercept plus FOLFIRI on patient quality of life (QoL) was not evaluated [12]. Second, when patients were enrolled in VELOUR, EGFR-I which represents the current standard of care for *RAS* wild-type tumors of the left colon in the first-line setting, were not yet available for use at the time of trial recruitment [13]. Thus, the activity of aflibercept plus FOLFIRI in patients previously treated with EGFR-I was unknown [12]. Moreover, the activity of aflibercept plus FOLFIRI according to *RAS* status was not reported in the VELOUR trial. The QoLiTrap study of the AIO working group “quality of life and patient-reported outcomes” was designed to address these important gaps in the data that may affect daily clinical practice.

## 2. Materials and Methods

### 2.1. Study Design

QoLiTrap was a prospective, multinational, multicenter, observational study that involved 408 sites across 3 countries (Austria, Germany, and Switzerland). QoLiTrap has been registered with the Arbeitsgemeinschaft Internistische Onkologie and given the identifier AIO-LQ-0113. Patients with mCRC who were eligible for treatment with aflibercept (trade name, Zaltrap^®^) plus FOLFIRI as per physician choice in daily practice, were enrolled in the study. Patients received the recommended dose of aflibercept (4 mg/kg of body weight), administered as an intravenous (iv) infusion over 1 h, followed by the FOLFIRI regimen (irinotecan 180 mg/m^2^ iv plus leucovorin 400 mg/m^2^ iv on day 1, followed by an iv bolus of [5-FU] 400 mg/m^2^ and a continuous iv infusion of 5-FU 2400 mg/m^2^ over 46 h). The treatment cycle was repeated every 2 weeks. There were no specifications regarding the number of cycles to be administered or any potential dose reductions or delays.

### 2.2. Patient Selection

Patients with mCRC who had progressed following an oxaliplatin-based regimen were eligible to enroll in the study as per the label. No further inclusion or exclusion criteria other than those specified in the indication for aflibercept were applied, reflecting real-world use.

### 2.3. Assessment

Since the study was conducted in daily practice, local guidelines of the respective clinics were applied. No further recommendations regarding patient monitoring, such as the frequency of follow-up visits and investigations to be performed at each visit, were provided. Patients who participated in the study agreed to take the European Organization for the Research and Treatment of Cancer Core Quality of Life Questionnaire (EORTC QLQ-C30), version 3.0, at baseline and at each visit during the treatment phase (generally bi-weekly). The EORTC QLQ-C30 measures 5 multi-item functional dimensions (physical, role, emotional, cognitive, and social), 3 multi-item symptoms (fatigue, nausea or vomiting, and pain), 6 single items (dyspnea, sleep disturbance, appetite loss, constipation, diarrhea, and financial impact), and 1 multi-item global health status (GHS) scale [14]. For functional and global QoL scales, higher scores represent improvements; for symptom scales, higher scores represent worsening symptoms [15].

All adverse events (AEs), serious and non-serious, related and not related, that occurred after the administration of the first dose of aflibercept until 28 days following administration of the last dose, were reported by German and Austrian sites. For Swiss sites, all adverse drug reactions causally related to aflibercept (serious and non-serious) were reported during the treatment period in accordance with local pharmacovigilance requirements. All AEs were coded according to the Medical Dictionary for Regulatory Activities, version 23.0 and summarized according to the National Cancer Institute Common Terminology Criteria for Adverse Events, version 4.0.

Tumor objective response rate (ORR) was defined as the proportion of patients with an investigator-assessed complete response (CR) or partial response (PR), based on imaging data, as the best response during therapy. A disease control rate (DCR) was defined as the proportion of patients with a CR, a PR, or stable disease (SD) as the best response during therapy. Only patients with documented imaging results were evaluated for ORR, and no central review of imaging was conducted. PFS was defined as the time from the first cycle initiation to the date of the first documented disease progression or death. OS was defined as the time from the initiation of the first cycle to the date of death from any cause.

### 2.4. Endpoints

The primary endpoint was the percentage of patients whose GHS either improved (≥0%) or was reduced by <5% from baseline during treatment with aflibercept plus FOLFIRI over a 12-week period. Secondary endpoints included ORR, PFS, OS, QoL subscales, and safety.

### 2.5. Statistical Analysis

The primary endpoint was analyzed in all patients having an evaluable QoL questionnaire at baseline and at least 2 evaluations post-baseline (QoL population). The ORR, DCR, PFS, and OS were analyzed with the intention to treat (ITT; all patients exposed to ≥1 cycle of aflibercept plus FOLFIRI). Safety was analyzed in the ITT population.

The QoLiTrap aimed to include approximately 1500 patients, to yield a target of 750 evaluable patients for the primary endpoint with a 95% CI of 46.4–53.6%. Analyses were descriptive and *p*-values were exploratory. No imputation of missing values was performed. Stratified log-rank tests were used to analyze time-to-event data. The Kaplan–Meier method was used to estimate survival curves, and the Cochran–Mantel–Haenszel chi-square tests were used to analyze categorical data.

The EORTC QLQ-C30 data were analyzed using the EORTC scoring manual, with scores ranging from 0 to 100 [14]. Descriptive statistics for EORTC QLQ-C30 were provided for all cycles for which the number of evaluable patients reached ≥20% vs. baseline. Factors influencing GHS were evaluated using multivariate stepwise logistic regression. Variables examined included ORR, hematological or nonhematological toxicity, pre-existing symptoms, baseline Eastern Cooperative Oncology Group performance status (ECOG PS; <2 vs. ≥2), and age (<60 vs. ≥60 years).

Primary and secondary endpoints were analyzed by *RAS* status (wild-type or mutant), sex, prior targeted therapy (bevacizumab and EGFR-I), and line of therapy. All analyses were performed at the cut-off date of 1 July 2020, using SAS software version 9.4 (SAS Institute Inc., Cary, NC, USA).

## 3. Results

### 3.1. Patient Characteristics

**Figure 1:**
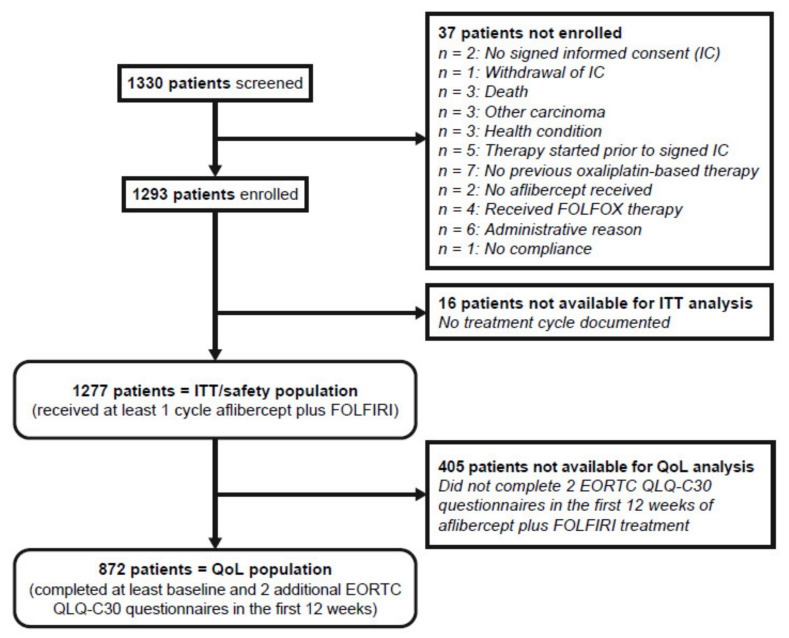
CONSORT diagram. EORTC QLQ-C30, European Organization for the Research and Treatment of Cancer Core Quality of Life Questionnaire; FOLFIRI, fluorouracil (5-FU), leucovorin, irinotecan; FOLFOX, fluorouracil (5-FU), leucovorin, oxaliplatin; ITT, intention to treat (population); QoL, Quality of life (population).

Between September 2013 and September 2019, 1330, patients with mCRC were screened, and 1293 patients were enrolled. Recruitment was stopped when the targeted number of evaluable patients for the primary endpoint was reached (Figure 1). Of the enrolled patients, 1277 (98.8%) received ≥1 cycle of aflibercept plus FOLFIRI, constituting the ITT or safety population, and 872 (67.4%) were evaluable for QoL analysis (QoL population; 1 evaluation at baseline and ≥2 evaluations post-baseline).

### 3.2. Baseline Characteristics and Treatment Exposure

Patient characteristics and disease history (including prior anti-cancer therapies) are illustrated in Table 1. Most patients exposed to aflibercept plus FOLFIRI were males (64.8%), 18.3% were aged ≥75 years, 69.8% had concomitant comorbidities (mainly cardiovascular), and 84.7% had an ECOG PS of 0–1 at treatment initiation. Tumor histology was adenocarcinoma in 96.2% of patients. Most tumors (69.3%) were left-sided; 50.7% of tumors contained RAS mutations, and metastases were located mainly in the liver (53.2%). Overall, 82.2% of patients had prior tumor surgery and 82.2% of patients had received prior targeted therapies (bevacizumab, 53.9%; EGFR-I, 13.2%; both, 15.1%) (Table 1). Baseline characteristics were similar between the ITT and QoL datasets.

Aflibercept plus FOLFIRI was prescribed in the second-line setting in 50.3% of patients; 56.9% of whom had *RAS* mutant and 43.5% *RAS* wild-type tumors (Supplementary Figure S1).

The median duration of treatment was 12 weeks, which corresponded to a median number of 6 cycles (range: 1–66). Overall, 10,197 cycles were administered. Dose modifications or delays were documented in 6.3% of patients; the incidence of dose changes increased with the increasing number of cycles received. The main reasons for treatment discontinuation were disease progression (44.2%), adverse events (21.2%), and patient requests (14.4%).

### 3.3. Primary Endpoint

**Figure 2:**
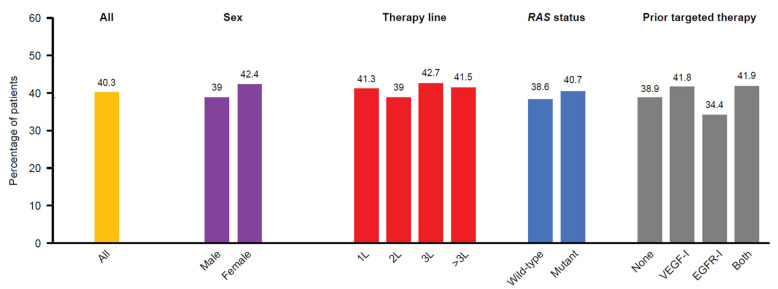
Percentage of patients with global health status that was stable or decreased by less than 5% from baseline within the first 12 weeks of treatment by sex, therapy line, *RAS* status, and prior targeted therapy. 1L, first-line; 2L, second-line; 3L, third-line; EGFR-I, epidermal growth factor receptor inhibitors; VEGF-I, vascular endothelial growth factor inhibitors.

EORTC QLQ-C30 was evaluable in 872 (68.3%) patients treated with aflibercept plus FOLFIRI. Completion compliance for EORTC QLQ-C30 for each treatment cycle is provided in Supplementary Table S1 and ranged from 68 to 77%. Overall, 351 patients (40.3%) had a GHS that improved or decreased by ≤5% throughout the first 12 weeks of treatment. Sex, *RAS* status, line of treatment, and prior targeted therapies had no impact on the GHS score (Figure 2).

#### EORTC QLQ-C30 Other Analyses

**Figure 3:**
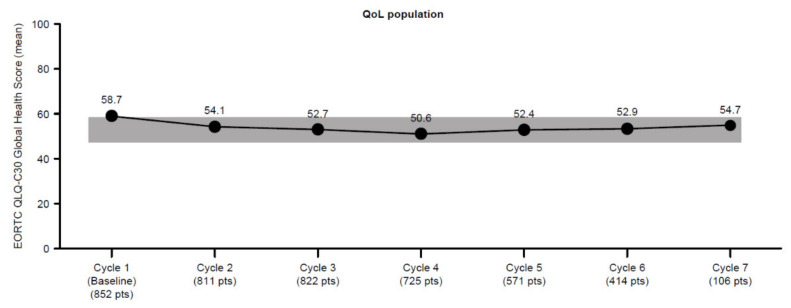
Mean global health status during the first seven cycles in the overall QoL population. Gray marking represents a clinically non-meaningful decline from the baseline value. 1L, first-line; 2L, second-line; 3L, third-line; EORTC QLQ-C30, European Organization for the Research and Treatment of Cancer Core Quality of Life Questionnaire; QoL, quality of life.

The baseline GHS was 58.7 and the maximum mean change from baseline was −4.6% within the first 12 weeks of treatment (Figure 3). The mean decline in GHS for patients with *RAS* mutant and *RAS* wild-type tumors was −6.0% from a baseline of 59.2 and −3.4% from a baseline of 58.2, respectively.

Changes in functional and symptom scales of EORTC QLQ-C30 for the overall population are shown until cycle 13 in Supplementary Figures S2 and S3. The largest decrease was observed in role functioning at cycle 4 (51.5 vs. 61.5 at baseline, mean values). Fatigue, sleep disturbances, pain, and appetite loss were the most prevalent symptoms at baseline. During treatment, a transient worsening of symptoms was observed, which was greatest at cycle 4 (Supplementary Figure S3). Similar findings were observed for the *RAS* wild-type and *RAS* mutant subgroups (data not shown).

Univariate and multivariate analyses showed that an ECOG PS ≥2 at baseline and the development of hematological toxicity during therapy (odds ratio, 4.94 and 1.71, respectively) were independently associated with an improved GHS at 12 weeks (Supplementary Table S2).

### 3.4. Secondary Endpoints

#### 3.4.1. Tumor Response

Tumor response was evaluated in 674 patients for whom imaging data were available. Overall, 7 patients (1.0%) had a CR, 133 patients (19.7%) had a PR, 326 patients (48.4%) were stable, and 193 patients (28.6%) experienced disease progression. Data were missing for 15 patients (2.2%). ORR was 20.8% and DCR was 70.0%. Tumor response rate was not affected by *RAS* status or sex. The highest values were observed in the first line setting (33.3%), in patients who had not received prior targeted therapies (28.9%), and those previously treated with EGFR-I (23.7%) (Table 2).

#### 3.4.2. Progression-Free Survival

**Figure 4:**
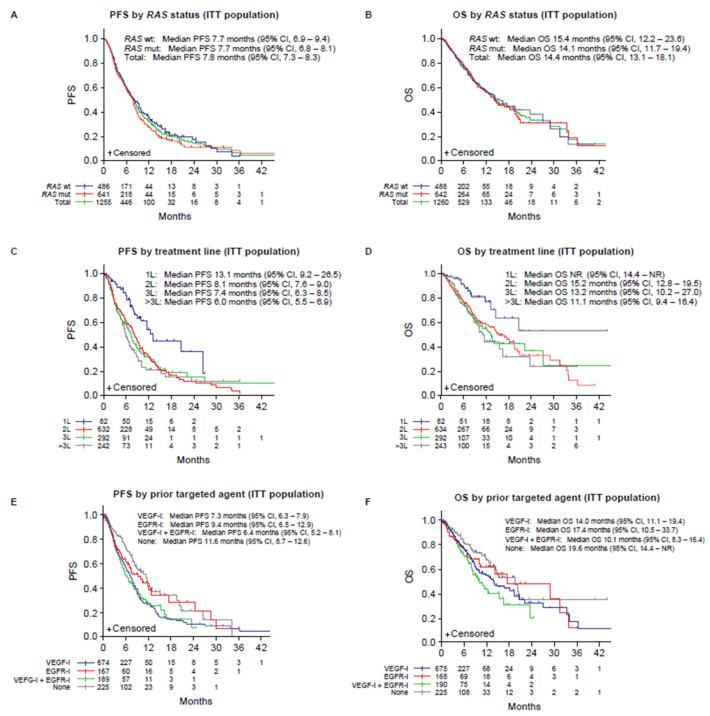
Kaplan–Meier estimates for PFS and OS according to *RAS* wild-type and *RAS* mutant patients (**A**,**B**), treatment line (**C**,**D**) and prior targeted therapy (**E**,**F**). EGFR-I, epidermal growth factor receptor inhibitors; CI, confidence interval; ITT, intention to treat (population); mutant; NR, not reported; OS, overall survival; PFS, progression-free survival; VEGF-I, vascular endothelial growth factor inhibitors; wt, wild-type.

A total of 617 patients experienced progression or death during the treatment period. Median PFS in the overall population was 7.8 months (95% CI, 7.3–8.3) (Figure 4, Table 2). The median PFS was not affected by *RAS* status or sex. Longer PFS values were observed in the first-line setting (13.1 months), in patients who had not received prior targeted therapies (11.6 months), and in those previously treated with EGFR-I (9.5 months) (Table 2).

#### 3.4.3. Overall Survival

During the treatment period, 363 deaths were reported. The median OS in the overall population was 14.4 months (95% CI, 13.1–18.1) (Figure 4, Table 2). Median OS was not impacted by *RAS* status but was slightly shorter in males than in females (14.2 vs. 19.4 months) and beyond third-line setting. Patients previously treated with EGFR-I had a longer OS than those previously treated with VEGF-I (17.4 vs. 14.0 months) (Figure 4, Table 2).

### 3.5. Subsequent Therapies following Aflibercept Plus FOLFIRI

Overall, a subsequent therapy following aflibercept plus FOLFIRI was documented in 821 patients (64.3%): chemotherapy plus anti-angiogenic agent (14.5%); chemotherapy plus EGFR-I (9.1%); FOLFOX (leucovorin, 5-FU, oxaliplatin; 6.7%); FOLFIRI (6.7%); 5-FU or capecitabine (25.2%); TAS-102 (trifluridine-tipiracil; 12.5%); regorafenib (11.9%); other (7.4%); best supportive care (6.0%).

### 3.6. Safety

AEs of any grade were reported in 1056 (82.7%) patients treated with aflibercept plus FOLFIRI. Grade ≥3 AEs were reported by 666 patients (52.2%) and serious AEs of any grade, regardless of causality, were reported by 559 patients (43.8%). Hypertension (9.3%), diarrhea (6.7%), general physical health deterioration due to disease progression (4.9%), stomatitis (3.9%), decreased leukocyte (3.4%), and neutrophil (2.3%) count were the most common grade ≥ 3 adverse events (Supplementary Table S3). Pulmonary embolism or embolism were reported by 2.7% of patients. One patient developed reversible posterior leukoencephalopathy syndrome.

Of, 1277 treated with aflibercept plus FOLFIRI, 185 (14.5%) died during the study. AEs leading to death were related mainly to disease progression (*n* = 69), worsening of general condition (*n* = 20), hepatic failure (*n* = 10), ileus (*n* = 9) or multiorgan dysfunction (*n* = 7). There were nine cases of sepsis, including one of neutropenic sepsis; four cases of hemorrhage (gastro-duodenal bleeding, *n* = 2; rectal tumor bleeding, *n* = 1; intracranial hemorrhage likely due to anticoagulation for atrial fibrillation, *n* = 1); and four cases of pulmonary embolism.

## 4. Discussion

Angiogenesis is an important therapeutic target for the treatment of mCRC [8,9,16] and aflibercept plus FOLFIRI is an established second-line treatment option in this setting, based on the VELOUR randomized phase III trial [12]. By prospectively documenting the QoL, effectiveness, and safety of aflibercept plus FOLFIRI in an unselected population treated in daily clinical practice, QoLiTrap complements the findings of the VELOUR trial [12]. Importantly, QoLiTrap confirms the activity of the regimen in patients previously treated with EGFR-I, which could not be explored at the time of VELOUR [13,17].

First, QoL was maintained in a high percentage of patients in this real-world population as aflibercept plus FOLFIRI showed no appreciative effect on GHS, as assessed by the EORTC QLQ-C30 [15]. Second, the results show that aflibercept plus FOLFIRI is prescribed mainly in the second-line setting in mCRC patients after the failure of an oxaliplatin-based regimen (50.3%), as per label and mCRC guidelines [8]. Third, the study confirms that aflibercept plus FOLFIRI exhibits a high tumor response rate (20.8%) and remains active regardless of *RAS* status, sex, and prior exposure to EGFR-I. Lastly, the safety profile of aflibercept plus FOLFIRI in an unselected population treated in daily clinical practice was consistent with the known safety profile of the regimen observed in previous trials. No new safety signals were identified.

Compared with the VELOUR study [12], the QoLiTrap population was older (median age, 66 vs. 61 years), had more comorbidities, and was more heavily pretreated. Indeed, 23.1% and 11.9% were treated in the third-line or beyond the third-line setting, respectively, whereas patients in VELOUR were treated exclusively in the second-line setting. Furthermore, patients in QoLiTrap exhibited a higher rate of ECOG PS 2 than those enrolled in VELOUR (6.7% vs. 2.2%). Regardless of these factors, QoLiTrap patients exhibited no clinically relevant decline of 10 points in EORTC QLQ-C30 GHS and subscale scores, even though they could be regarded as sicker [14]. QoLiTrap findings also appear to be in agreement with those of the Aflibercept Safety and health-related Quality-of-life Program (ASCoP) study, which enrolled 779 patients with mCRC exclusively treated in the second-line setting and who had to satisfy the same inclusion and exclusion criteria as those of VELOUR [18,19].

In QoLiTrap, the tumor response rate to aflibercept plus FOLFIRI was 20.8% in the overall population and reached 24% in patients treated in the second-line setting. These results are comparable to those observed in the VELOUR study (19.8% with aflibercept plus FOLFIRI vs. 11.1% with placebo plus FOLFIRI, *p* = 0.0001) [12]. In the Treatment Multiline (TML) study, which compared chemotherapy plus bevacizumab continuation versus chemotherapy alone in the second-line setting, the response rate was lower and similar between the treatment arms (6% vs. 4%) [20]. Similarly, in the RAISE study, which compared ramucirumab plus FOLFIRI versus placebo plus FOLFIRI in the second-line setting, no difference in the response rate was observed between the treatment arms (13.4% vs. 12.5%) [21]. Retrospective studies comparing FOLFIRI plus bevacizumab continuation versus FOLFIRI plus aflibercept in second-line settings are difficult to interpret, since patients receiving aflibercept usually had more aggressive features [22,23,24]. For example, in a retrospective study that included 681 mCRC patients treated in a second-line setting, FOLFIRI plus aflibercept was associated with a comparable objective response rate (20.6% vs. 25.6%), a shorter PFS (5.1 vs. 6.0 months), and OS (10.4 vs. 13.0 months) compared to FOLFIRI plus bevacizumab. However, the percentage of patients with *RAS* and *BRAF* wild-type tumors, known to have a better prognosis, was significantly lower in the aflibercept group than in the bevacizumab group (8.12% vs. 20.53%, *p* = 0.001) [24]. Randomized clinical trials are thus needed to clarify the issue.

*RAS* mutations are a key molecular feature of mCRC and are associated with a worse prognosis and resistance to EGFR-I [8,25,26,27]. In the QoLiTrap study, patients with *RAS* mutations treated with aflibercept plus FOLFIRI achieved outcomes comparable to *RAS* wild-type tumors with respect to tumor response rate (21.3% in *RAS* mutant vs. 20.8% in *RAS* wild-type), PFS (7.7 vs. 7.7 months), and OS (14.1 vs. 15.4 months). EGFR-I has become the preferred first-line treatment option for patients with *RAS* wild-type tumors of the left colon [8]. During recruitment for the VELOUR study, this therapeutic class was not yet available [13]. Therefore, the activity of aflibercept plus FOLFIRI in patients previously treated with EGFR-I was unknown. The results of QoLiTrap may reassure physicians that aflibercept plus FOLFIRI retains its activity in patients previously treated with EGFR-I with a response rate of 23.7%, a PFS of 9.5 months, and an OS of 17.4 months.

Our results are in line with the known literature. In the subgroup analysis of the RAISE study, patients with wild-type *KRAS* tumors previously treated with oxaliplatin, bevacizumab, and fluoropyrimidine analogues showed a better PFS (5.7 vs. 4.7 months) and an OS (14.4 vs. 11.9 months) with FOLFIRI plus ramucirumab as compared with FOLFIRI plus placebo [28]. In the randomized phase II trial, Partenariat de Recherche en Oncologie DIGEstive 18 (PRODIGE 18), which compared continued bevacizumab versus cetuximab in the second-line treatment of *RAS* wild-type tumors, median PFS and OS with bevacizumab versus cetuximab were 7.8 vs. 5.6 months and 21 vs. 10.7 months, respectively [29]. In a retrospective study that included 277 patients with *RAS* wild-type tumors treated with EGFR-I in the first-line, followed by an anti-angiogenic therapy (82% received bevacizumab and 18% received aflibercept), the median PFS from the initiation of the second-line treatment was 7.1 months and the median OS was 15.7 months [30]. Findings from QoLiTrap suggest that mCRC patients previously treated with EGFR-I may also benefit from treatment with aflibercept plus FOLFIRI.

No new safety signals were observed in QoLiTrap. The most frequently reported AEs in the study were diarrhea, nausea, stomatitis, and hypertension, which are consistent with previous findings [12,18,31]. Despite a lack of exclusion criteria, together with the increased age, number of comorbidities, and prior treatments of enrolled patients, the results of QoLiTrap confirm that aflibercept plus FOLFIRI has a manageable safety profile even in this higher-risk population.

Our study has several limitations. First, because QoLiTrap was a prospective observational study that evaluated the daily practice of physicians, enrolled patients were more heterogeneous than those in randomized clinical trials [32]. Second, the timing of follow-up visits and tumor assessments was not prespecified, and there was no central review of imaging. However, a recent meta-analysis of randomized phase III trials disclosed no difference in PFS evaluated by the investigators or centrally reviewed [33]. This reinforces the validity of our results compared to the VELOUR study: tumor response (20.8% vs. 19.8%), PFS (7.76 vs. 6.90 months) and OS (14.4 vs. 13.5 months) [12]. Third, because the protocol requested an evaluable QoL, questionnaire at baseline and at least 2 questionnaires post-baseline, only 872 out of 1277 patients exposed to aflibercept plus FOLFIRI (67.4%) were analyzed for QoL which represents an important bias. Lastly, the results of laboratory tests were not recorded and angiogenic biomarkers (PlGF, VEGF-A) were not analyzed, precluding a comparison with the VELOUR trial.

## 5. Conclusions

In summary, this prospective study conducted in daily practice suggests that patients with mCRC treated with aflibercept plus FOLFIRI maintain their quality of life, as assessed by the EORTC QLQ-C30 questionnaire. Aflibercept plus FOLFIRI was also associated with a high objective tumor response and retained its activity regardless of sex, *RAS* status, and prior targeted therapy, especially after EGFR-I. Adverse events were consistent with the known safety profile of aflibercept plus FOLFIRI.

## Figures and Tables

**Table 1:** Baseline characteristics of patients enrolled in the ITT and QoL datasets.

Baseline Characteristic	ITT/Safety Set (*n* = 1277)	QoL Set (*n* = 872)
Sex, *n* (%)		
Male	827 (64.8)	561 (64.3)
Female	450 (35.2)	311 (35.7)
Age, years		
Median (range)	66 (28–90)	66 (28–88)
<70, *n* (%)	785 (61.5)	564 (64.7)
70 < 75, *n* (%)	259 (20.3)	165 (18.9)
≥75, *n* (%)	233 (18.3)	143 (16.4)
Comorbidities, *n* (%)		
Cardiovascular	625 (48.9)	411 (47.1)
Diabetes	210 (16.4)	134 (15.4)
Dyslipidemia	67 (5.2)	39 (4.5)
Kidney or liver	79 (6.2)	48 (5.5)
disorders		
Lung disorders	77 (6.0)	51 (5.8)
ECOG, *n* (%)		
0–1	1082 (84.7)	761 (87.3)
2–3	85 (6.7)	37 (4.2)
Missing	110 (8.6)	74 (8.5)
Histology, *n* (%)		
Adenocarcinoma	1228 (96.2)	841 (96.4)
Other	9 (0.7)	6 (0.7)
N/A	40 (3.1)	25 (2.9)
Tumor location, *n* (%)		
Right colon	352 (27.6)	245 (28.1)
Left colon	885 (69.3)	607 (69.6)
Metastatic sites, *n* (%)		
Liver	679 (53.2)	475 (54.5)
Lung	220 (17.2)	161 (18.5)
Peritoneum	156 (12.2)	108 (12.4)
Lymph nodes	143 (11.2)	93 (10.7)
Other	60 (4.7)	37 (4.2)
*RAS* status, *n* (%)		
*RAS* wild-type	497 (38.9)	339 (38.9)
*RAS* mutant	648 (50.7)	450 (51.6)
N/A	132 (10.3)	83 (9.5)
Prior targeted therapy, *n* (%)		
VEGF-I	688 (53.9)	469 (53.8)
EGFR-I	168 (13.2)	122 (14.0)
VEGF-I + EGFR-I	193 (15.1)	124 (14.2)
None	228 (17.9)	157 (18.0)

ECOG, Eastern Cooperative Oncology Group; EGFR-I, epidermal growth factor receptor inhibitors; ITT, intention to treat (population); NA, not available; QoL, quality of life; VEGF-I, vascular endothelial growth factor inhibitors.

**Table 2:** Overall survival, progression-free survival, and tumor response rate of aflibercept plus FOLFIRI in the ITT population, stratified by subgroups.

		OS, Months		PFS, Months		Tumor Response, *n* (%)		
	*N*	Median	95% CI	Median	95% CI	*N*	CR + PR	DCR
Overall	1265	14.4	13.1–18.1	7.8	7.3–8.3	674	140 (20.8)	466 (70.0)
*RAS* status								
*RAS* wt	492	15.4	12.2–23.6	7.7	6.9–9.4	250	52 (20.8)	175 (69.1)
*RAS* mutant	645	14.1	11.7–19.4	7.7	6.8–8.1	362	77 (21.3)	21 (66.6)
Sex								
Male	820	14.2	12.5–16.8	7.9	7.3–8.9	432	93 (21.5)	304 (70.4)
Female	445	19.4	11.8–23.6	7.5	6.4–8.3	242	47 (19.4)	162 (66.9)
Therapy line								
1L	82	NR	14.4–NR	13.1	9.2–26.5	48	16 (33.3)	42 (87.5)
2L	636	15.2	12.8–19.5	8.1	7.6–9.0	350	84 (24.0)	255 (72.9)
3L	293	13.2	10.2–27.0	7.4	6.3–8.5	143	22 (15.4)	89 (62.2)
>3L	247	11.1	9.4–16.4	5.9	5.5–6.9	130	17 (13.1)	77 (59.2)
Prior targeted therapy								
None	225	19.6	14.4-NR	11.6	8.7–12.6	114	33 (28.9)	93 (81.6)
EGFR-I	168	17.4	10.5–33.7	9.5	6.5–12.9	93	22 (23.7)	73 (78.5)
VEGF-I	681	14.0	11.1–19.4	7.3	6.3–7.9	376	73 (19.4)	245 (65.2)
Both	191	10.1	8.3–16.4	6.2	5.2–8.1	91	12 (13.2)	55 (60.4)

1L, first-line; 2L, second-line; 3L, third-line; EGFR, epidermal growth factor receptor inhibitors; CI, confidence interval; CR, complete response; DCR; disease control rate; *N*, number of patients; NR, not reported; OS, overall survival; PFS, progression-free survival; PR, partial response; VEGF, vascular endothelial growth factor inhibitors; wt, wild-type.

## Data Availability

Qualified researchers may request access to patient-level data and related study documents including the clinical study report, study protocol with any amendments, blank case report form, statistical analysis plan, and dataset specifications. Patient-level data will be anonymized, and study documents will be redacted to protect the privacy of our trial participants. Further details on Sanofi’s data sharing criteria, eligible studies, and process for requesting access can be found at: https://www.vivli.org/ (accessed on 16 June 2022).

## Supplementary Materials

The following supporting information can be downloaded at: https://www.mdpi.com/article/10.3390/cancers14143522/s1, Figure S1: Percentage of patients receiving aflibercept in first-line, second-line, third-line, fourth-line, and fifth-line setting, in the ITT population and by RAS status; Figure S2: Changes in EORTC QLQ-C30 functioning scales from baseline to cycle 13: (A) Physical functioning, (B) Role functioning, (C) Emotional functioning, (D) Cognitive functioning, and (E) Social functioning; Figure S3: Changes in EORTC QLQ-C30 symptom scales from baseline to cycle 13: (A) Fatigue, (B) Pain, (C) Dyspnea, (D) Sleep disturbance, (E) Appetite loss, (F) Constipation, (G) diarrhea, (H) Financial impact, and (I) Nausea or vomiting; Table S1: Patient disposition in terms of completion of EORTC QLQ-C30; Table S2: Factors associated with an improved GHS score, as assessed by univariate (2A) and multivariate (2B) analysis; Table S3: Adverse events that occurred in ≥3% of patients treated with aflibercept plus FOLFIRI (n = 1277); Table S4: List of QoLiTrap investigators.
