# Supplementary material for: Real-World Evaluation of Quality of Life, Effectiveness, and Safety of Aflibercept Plus FOLFIRI in Patients with Metastatic Colorectal Cancer: The Prospective QoLiTrap Study
Source: Cancers (Basel). 2022 Jul 20;14(14):3522. doi: 10.3390/cancers14143522 (PMC9324206; doi:10.3390/cancers14143522)
Supplement: Supplementary file 1 [file cancers-14-03522-s001.zip › Supplementary Tables.pdf]

Supplementary Table S1. Patient disposition in terms of completion of EORTC QLQ-C30. EORTC QLQ-C30, European Organization for the Research and Treatment of Cancer Core Quality of Life Questionnaire.

**Cycle number**	**Number of patients who completed the questionnaire per cycle, *n/N (%)*** **(*N*=1277)**
Cycle 1 (baseline)	852/1276 (66.8)
Cycle 2	811/1178 (68.8)
Cycle 3	822/1070 (76.8)
Cycle 4	731/946 (77.3)
Cycle 5	615/819 (75.1)
Cycle 6	546/718 (76.0)
Cycle 7	422/576 (73.3)
Cycle 8	373/483 (77.2)
Cycle 9	310/421 (73.6)
Cycle 10	281/384 (75.0)
Cycle 11	240/321 (76.0)
Cycle 12	203/279 (72.8)
Cycle 13	161/217 (74.2)

Supplementary Table S2. Factors associated with an improved GHS score, as assessed by univariate (2A) and multivariate (2B) analysis. ECOG PS, Eastern Cooperative Oncology Group performance status; GHS, global health status.

2A Univariate analysis

Parameter	Odds Ratio	95% CI	*p*-value
ECOG PS			
0-1	Ref	-	-
≥ 2	6.57	[4.11 - 10.48]	<0.0001
Hematological toxicity			
No	Ref	-	-
Yes	3.79	[2.86 - 5.01]	<0.0001
Objective tumor response			
No	Ref	-	-
Yes	2.47	[1.76 - 3.48]	<0.0001
Preexisting symptoms			
No	Ref	-	-
Yes	0.93	[0.68 - 1.23]	<0.0001
Non-hematological toxicity			
No	Ref	-	-
Yes	0.85	[0.65 - 1.11]	0.22
Age			
< 60 years	Ref	-	-
≥ 60 years	0.52	[0.40 – 0.68]	<0.0001

2B multivariate analysis

Parameter	Odds Ratio	95% CI	*p*-value
ECOG PS			
0-1	Ref	-	-
≥ 2	4.94	[2.60 - 9.41]	0.0020
Hematological toxicity			
No	Ref	-	-
Yes	1.71	[1.14 - 2.55]	0.0095

Supplementary Table S3. Adverse events that occurred in ≥3% of patients treated with aflibercept plus FOLFIRI (n=1277). FOLFIRI, fluorouracil (5-FU), folinic acid, irinotecan.

**Patients**	**All grades, *n* (%)**	**Grade ≥ 3, *n* (%)**
Any adverse event	1056 (82.7)	666 (52.2)
Diarrhea	437 (34.2)	86 (6.7)
Fatigue/ asthenia	227 (17.8)	18 (1.4)
Nausea	228 (17.9)	14 (1.1)
Stomatitis	219 (17.2)	50 (3.9)
Hypertension	148 (11.6)	118 (9.3)
Decreased white blood cell count	116 (9.1)	43 (3.4)
General physical health deterioration	106 (8.3)	62 (4.9)
Weight loss	98 (7.7)	2 (0.2)
Decreased appetite	94 (7.4)	8 (0.6)
Vomiting	93 (7.3)	12 (0.9)
Abdominal pain	91 (7.1)	14 (1.1)
Dyspnea	84 (6.6)	20 (1.6)
Anemia	58 (4.8)	17 (1.3)
Peripheral sensory neuropathy	54 (4.2)	3 (0.2)
Neutrophil count decreased	54 (4.2)	30 (2.3)
Platelet count decreased	54 (4.2)	10 (0.8)
Pyrexia	53 (4.2)	4 (0.3)
Headache	51 (4.0)	2 (0.2)
Constipation	48 (3.8)	2 (0.2)
Epistaxis	45 (3.5)	1 (0.1)
Pain	43 (3.4)	4 (0.4)
Proteinuria	43 (3.4)	9 (0.7)
Dehydration	42 (3.3)	16 (1.3)
Back pain	39 (3.1)	7 (0.5)
Neoplasm progression	41 (3.2)	39 (3.1)
Dysphonia	39 (3.1)	3 (0.2)

Supplementary Table S4

The names of the QoLiTrap investigators, listed by country.

Last name	First name	Country
Enökl-Tomantschger	Ute	Austria
Greil	Richard	Austria
Grünberger	Birgit	Austria
Korger	Markus	Austria
Meran	Johannes	Austria
Muellner-Ammer	Kirsten	Austria
Popescu	Razvan	Austria
Prager	Gerald	Austria
Rossmann	Dieter	Austria
Thaler	Josef	Austria
Tinchon	Christoph	Austria
Abenhardt	Wolfgang	Germany
Anhut	Peter	Germany
Assmann	Michael	Germany
Baake	Gerold	Germany
Balleisen	Jan Sebastian	Germany
Balser	Christina	Germany
Banhardt	Ulrich	Germany
Basovski	Leonid	Germany
Becker	Cornelia	Germany
Behringer	Dirk	Germany
Benz	Jutta	Germany
Bergmann	Friedrich	Germany
Bischoff	Marina	Germany
Blau	Wolfgang	Germany
Blumenstengel	Klaus	Germany
Bolling	Claus	Germany
Brandl	Stephan	Germany
Bremer	Anne	Germany
Brinkmann	Lutz	Germany
Broszeit-Luft	Stefanie	Germany
Burkhard-Meier	Ulrike	Germany
Bürkle	Dieter	Germany
Däßler	Klaus-Ulrich	Germany
Denzlinger	Claudio	Germany
Depenbusch	Reinhard	Germany
Derigs	H. Guenter	Germany
Diener	Christian	Germany
Dresemann	Gregor	Germany
Dworzanski	Regina	Germany
Edelmann	Thomas	Germany
Egert	Matthias	Germany
Eggers	Egbert	Germany
Ehrig	Johannes	Germany
Eichstaedt	Martina	Germany

Elsel	Wolfgang	Germany
Eschenburg	Henning	Germany
Fechner	Lars	Germany
Fenchel	Klaus	Germany
Flieger	Dimitri	Germany
Fries	Stefan	Germany
Fritz	Markus	Germany
Fronhoffs	Stefan	Germany
Fuchs	Roswitha	Germany
Gabius	Sigrun	Germany
Garlipp	Benjamin	Germany
Gaska	Tobias	Germany
Geer	Thomas	Germany
Gerl	Arthur	Germany
Giagounidis	Aristoteles	Germany
Göhler	Thomas	Germany
Golf	Alexander	Germany
Graf	Nicolas	Germany
Groschek	Matthias	Germany
Grundeis	Marc	Germany
Grunewald	Ralf	Germany
Güller	Ulrich	Germany
Haas	Siegfried	Germany
Hacker	Ulrich	Germany
Haessner	Joachim	Germany
Hahn	Lars	Germany
Hansen	Richard	Germany
Hartmann	Frank	Germany
Heinrich	Bernhard Jürgen	Germany
Helbling	Daniel	Germany
Hempel	Dirk	Germany
Herb	Siegbert	Germany
Hering-Schubert	Christiane	Germany
Hessling	Jörg	Germany
Höblinger	Aksana	Germany
Höffkes	Heinz-Gert	Germany
Hoffmann	Thomas	Germany
Illerhaus	Gerald	Germany
Immenschuh	Peter	Germany
Jäckle	Jürgen	Germany
Jacobasch	Lutz	Germany
Janssen	Jan	Germany
Josting	Andreas	Germany
Just	Marianne	Germany
Käfer	Gabriele	Germany
Keitel-Anselmino	Verena	Germany
Kessler	Christina	Germany
Klaproth	Holger	Germany
Klausmann	Martine	Germany
Kleiss	Mathias	Germany

Klump	Bodo	Germany
Ko	Yon-Dschun	Germany
Koch	Bernhard	Germany
Koenigsmann	Michael	Germany
Kohl	Detlev	Germany
Krammer-Steiner	Beate	Germany
Kreher	Stephan	Germany
Kretzschmar	Albrecht	Germany
Kröning	Hendrik	Germany
Kühn	Gunhild	Germany
Kullmann	Frank	Germany
Lamberti	Christof	Germany
Lebahn	Herbert	Germany
Lebahn	Herbert	Germany
Lehenbauer-Dehm	Silvia	Germany
Leithäuser	Malte	Germany
Limmroth	Christina	Germany
Losem	Christoph	Germany
Lüdtke-Heckenkamp	Kerstin	Germany
Maier-Bay	Birgit	Germany
Mainka	Dieter	Germany
Maintz	Christoph	Germany
Maintz	Oliver	Germany
Martin	Kerstin	Germany
Maschmeyer	Georg	Germany
Mayer	Frank	Germany
Maywald	Ole	Germany
Menzel	Josef	Germany
Meyer	Dirk	Germany
Michl	Gerlinde	Germany
Moebius	Elke	Germany
Mohm	Johannes	Germany
Möller	Marcel	Germany
Müller	Gudrun	Germany
Müller	Christian	Germany
Musch	Reinhard	Germany
Neise	Michael	Germany
Oetzel	Carsten	Germany
Öhler	Leopold	Germany
Ohmenhäuser	Axel	Germany
Opitz	Bernhard Martin	Germany
Otremba	Burkhard	Germany
Papke	Jens	Germany
Petersen	Volker	Germany
Peuser	Bettina	Germany
Pistorius	Uwe	Germany
Rauh	Jacqueline	Germany
Ritter	Ute	Germany
Roitner	Florian	Germany
Rubanov	Oleg	Germany

Rudi	Jochen	Germany
Ruffert	Klaus	Germany
Sahm	Stephan	Germany
Salat	Christoph Tore	Germany
Sandner	Reiner	Germany
Sauer	Annette	Germany
Schaich	Markus	Germany
Schardt	Christof	Germany
Schauenberg	Philipp	Germany
Scheuer	Burkhard	Germany
Schmid	Mathias	Germany
Schmitt	Karla	Germany
Schmitz	Marion	Germany
Schmitz	Stephan	Germany
Schröder	Jan	Germany
Schröder	Detlev	Germany
Schubring	Markus	Germany
Schüler	Frank	Germany
Schulz	Holger	Germany
Schulz-Abelius	Armin	Germany
Schulze	Mathias	Germany
Schulze-Bergkamen	Henning	Germany
Schütz	Stefan	Germany
Schwarz	Maik	Germany
Schweigert	Marcus	Germany
Schweinitz	Cornelia	Germany
Schweizer	Christof	Germany
Schwerdtfeger	Michael	Germany
Schwinger	Ulrike	Germany
Schwittay	Michael	Germany
Seipelt	Gernot	Germany
Siegler	Gabriele	Germany
Söling	Ulrike	Germany
Spohn	Claudia	Germany
Stauch	Martina	Germany
Stern	Sieglinde	Germany
Tebbe	Sandra	Germany
Tischbirek	Klaus	Germany
Titgemeyer	Jan	Germany
Tschechne	Barbara	Germany
Uhlig	Jens	Germany
Valdix	Annette-Rosel	Germany
Vehling-Kaiser	Ursula	Germany
Waberzeck	Brigitte	Germany
Welslau	Manfred	Germany
Whitlock	Bettina	Germany
Wiegand	Jörg	Germany
Wilke	Jochen	Germany
Wolff	Thomas	Germany
Wortmann	Anke	Germany

Wullstein-Winkler	Felix	Germany
Wuttke	Patricia	Germany
Zahn	Mark-Oliver	Germany
Zander	Ingo	Germany
Zeth	Matthias	Germany
Ziske	Carsten	Germany
Anchisi	Sandro	Switzerland
Betticher	Daniel	Switzerland
Bohanes	Pierre	Switzerland
Kühne	Reto	Switzerland
Moosmann	Peter	Switzerland
Pederiva	Stefanie	Switzerland
von Moos	Roger	Switzerland
Zenhäusern	Reinhard	Switzerland
