# Supplementary figures and images for: Real-World Evaluation of Quality of Life, Effectiveness, and Safety of Aflibercept Plus FOLFIRI in Patients with Metastatic Colorectal Cancer: The Prospective QoLiTrap Study
Source: Cancers (Basel). 2022 Jul 20;14(14):3522. doi: 10.3390/cancers14143522 (PMC9324206; doi:10.3390/cancers14143522)

**Supplementary Figure S1**

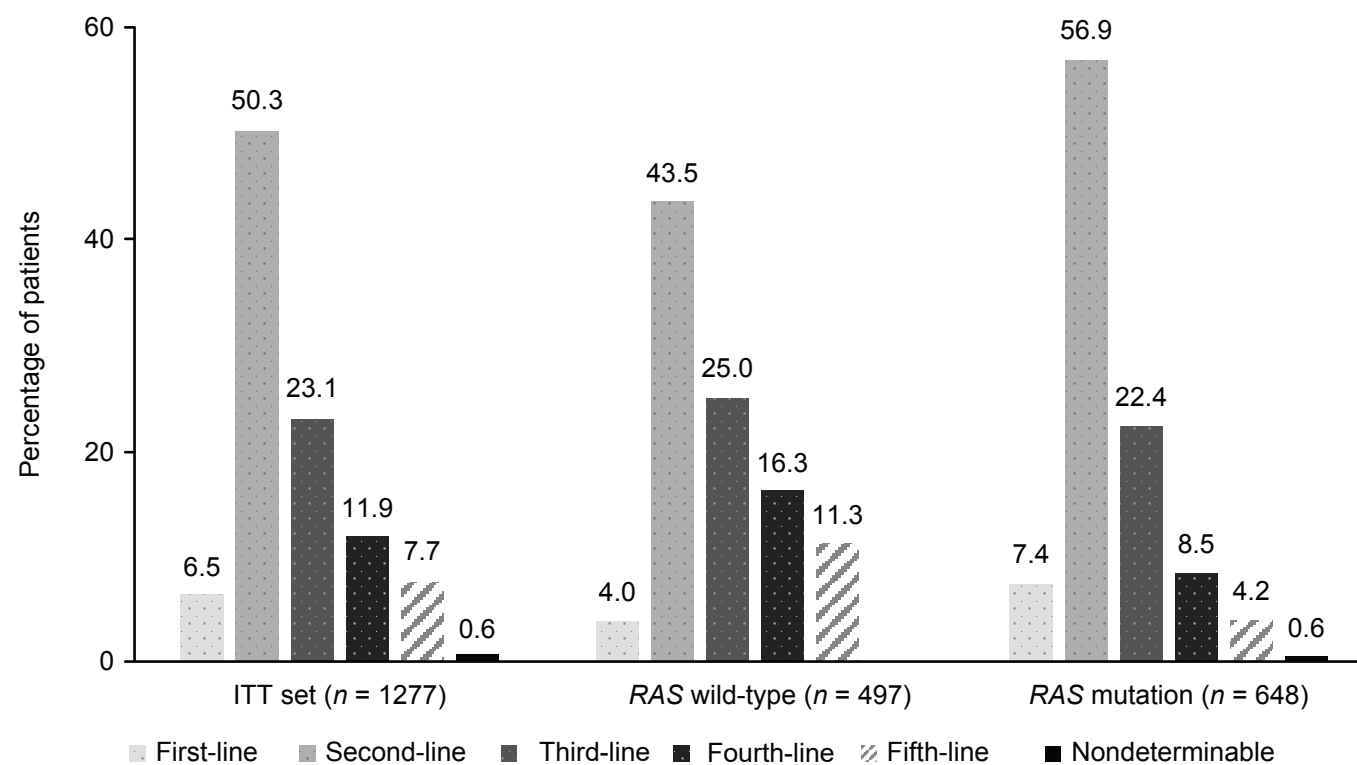

## Supplementary Figure S2

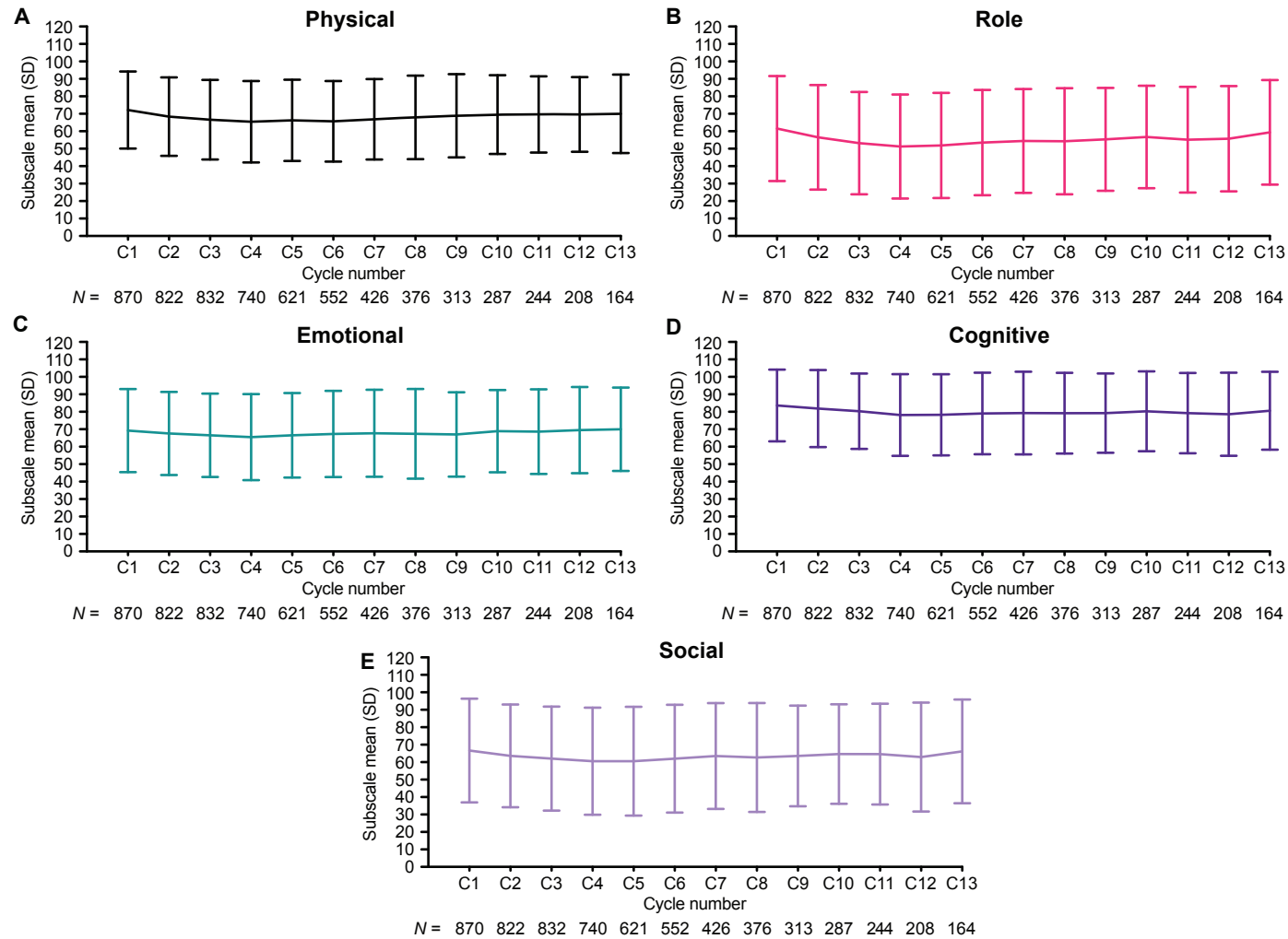

**Supplementary Figure S3**

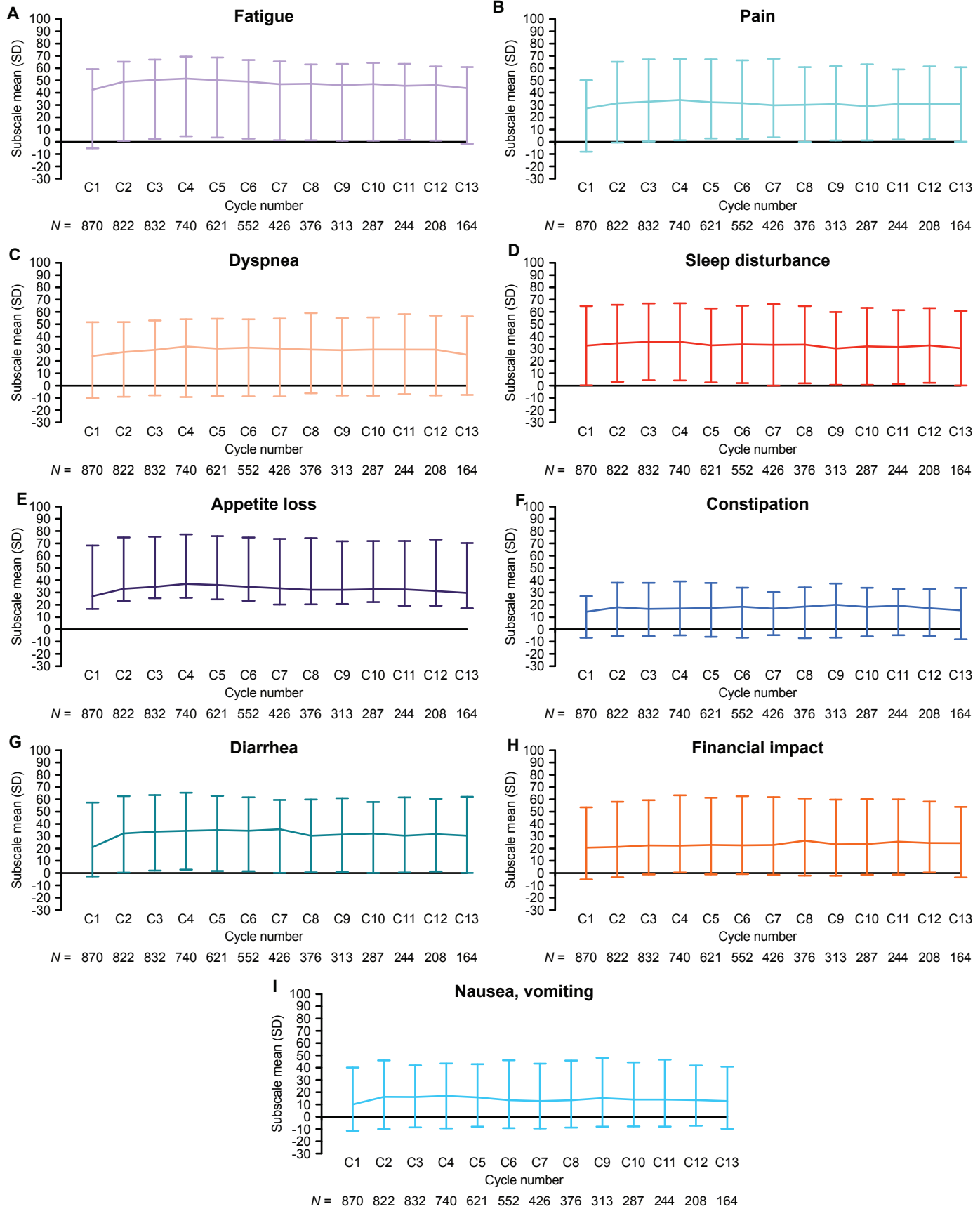

Supplement: Supplementary file 1 [file cancers-14-03522-s001.zip › Supplementary Figures.pdf]
